# Wnt Signaling Pathway: Biological Function, Diseases, and Therapeutic Interventions

**DOI:** 10.1002/mco2.70580

**Published:** 2026-01-14

**Authors:** Xiaoyu Jin, Jiahui Wang, Runyi Cao, Dongsheng Jiang

**Affiliations:** ^1^ Precision Research Center For Refractory Diseases Shanghai General Hospital Shanghai Jiao Tong University School of Medicine Shanghai China; ^2^ Trauma Center Shanghai General Hospital Shanghai Jiao Tong University School of Medicine Shanghai China; ^3^ State Key Laboratory of Innovative Immunotherapy School of Pharmacy Shanghai Jiao Tong University Pioneer Research Institute For Molecular and Cell Therapies Shanghai Jiao Tong University Shanghai China

**Keywords:** fibrosis, myofibroblast activation, secreted frizzled‐related protein (SFRP), therapeutic targeting, Wnt signaling

## Abstract

The Wnt signaling pathway deeply participates in multiple physiological and pathological processes. Its activity is intricately regulated by a diverse network of modulators, reflecting the pathway's structural and functional complexity. Dysregulation of Wnt signaling leads to cellular dysfunction and is associated with a wide spectrum of diseases, among which tissue fibrosis represents a major pathological outcome, characterized by activation of myofibroblasts and subsequent excessive deposition of extracellular matrix in response to injury. Wnt signaling is a central driver of fibrotic progression across multiple tissues and organs; however, effective therapeutic strategies directly targeting Wnt signaling in fibrosis remain scarce. In this review, we provide a comprehensive overview of Wnt pathway components, regulatory mechanisms, and therapeutic approaches. We systematically examine how Wnt signaling governs both developmental processes and pathological conditions, with particular emphasis on its role in fibrosis while also extending discussion to other diseases. Special attention is devoted to the secreted frizzled‐related proteins (SFRPs) family, soluble regulators with biphasic, context‐dependent effects that are especially relevant in fibrosis. Finally, we summarize insights from preclinical and clinical studies, review advances and challenges in the development of small‐molecule compounds targeting Wnt components, highlighting the vital role of SFRPs as promising targets for antifibrotic intervention.

## Graphical abstract

The Wnt signaling pathway is essential for development and tissue homeostasis, while its dysregulation drives diverse diseases. This review systematically outlines its components, functions, regulators, and preclinical models, highlighting secreted frizzled‐related proteins (SFRPs) as context‐dependent, biphasic modulators. Their dual roles in tissue fibrosis underscore both the complexity of Wnt regulation and the therapeutic potential of SFRPs.

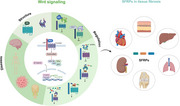

## 1 Introduction

The Wnt signaling pathway is a complex and evolutionarily conserved network that orchestrates numerous biological processes, from embryonic patterning to adult tissue homeostasis [1, 2, 3]. The Wnt1 gene, originally named Int‐1, was first identified in mice in 1982, as the first member of the Wnt family [4]. Shortly thereafter, its homolog in Drosophila, the Wingless (Wg) gene, was found to control segment polarity during larval development [5, 6]. Since then, key components of the core developmental Wnt signaling cascade, including porcupine, dishevelled (Dvl), armadillo (Arm) (β‐catenin), and zeste‐white 3/glycogen synthase kinase (GSK)3 gene, as well as T cell factor/lymphoid enhancer factor (TCF/LEF) transcription factors, frizzleds (Fz) as Wnt receptors, and LRPs/Arrow as coreceptors, have been discovered one after another [7, 8, 9]. The first link between Wnt signaling and human disease was established with familial adenomatous polyposis, a cancer caused by mutations in the adenomatous polyposis coli (APC) gene [10]. The Wnt pathway has been recognized as a fundamental regulator of cell fate determination, proliferation, migration, and polarity [11]. It operates primarily through two major branches: the canonical (β‐catenin‐dependent) pathway and the noncanonical (β‐catenin‐independent) pathways, which include the Wnt/planar cell polarity (PCP) and Wnt/calcium (Wnt/Ca^2^⁺) pathway [12, 13, 14]. Together, these signaling modes control a wide range of cellular and developmental events and are spatiotemporally regulated to ensure normal physiology.

The canonical Wnt/β‐catenin pathway regulates transcriptional programs essential for early embryogenesis [15], tissue morphogenesis [16], and adult stem cell maintenance in regenerative niches such as the intestinal crypts and hair follicles [1, 17, 18, 19]. In contrast, noncanonical pathways function independently of β‐catenin to coordinate tissue architecture and dynamic cellular behaviors. The PCP pathway governs cell polarity, directional migration, and oriented cell division, thereby ensuring proper cell alignment and tissue organization [20, 21, 22]. The Wnt/Ca^2+^ pathway modulates intracellular calcium signaling, influencing processes such as cytoskeletal remodeling, cell adhesion, and motility [23, 24, 25]. Dysregulation of these pathways has been implicated in a broad spectrum of diseases, including developmental disorders, cancer, degenerative diseases, and fibrosis.

Fibrosis represents a pathological endpoint of chronic injury characterized by persistent inflammation, fibroblast activation, and excessive deposition of extracellular matrix (ECM). Affecting organs such as the lung, liver, kidney, heart, and skin, fibrosis progressively disrupts tissue structure and function, leading to irreversible organ failure. In addition to the classical transforming growth factor (TGF)‐β pathway, Wnt signaling has emerged as a key driver of fibrotic remodeling, influencing fibroblast differentiation, ECM production, and crosstalk with microenvironment cells and other pathways [26, 27, 28, 29, 30]. Among the multiple regulators of Wnt signaling, secreted frizzled‐related proteins (SFRPs) have attracted increasing attention [31, 32, 33, 34].

In this review, we provide an overview of the Wnt signaling pathway in human health and disease, with emphasis on its biological functions, roles in major pathological conditions, and therapeutic potential. Particular focus is placed on the SFRP family as modulators of Wnt activity and their contribution to tissue fibrosis. We outline the molecular framework of Wnt signaling and its crosstalk with other pathways, examine its relevance across disease categories with a special focus on fibrosis, and highlight therapeutic strategies targeting Wnt signaling, especially through SFRPs, before offering future perspectives.

## 2 Wnt Signaling: Structure and Modes of Action

Wnt signaling is built upon a highly coordinated molecular framework in which ligand–receptor interactions initiate a spectrum of intracellular responses that govern cellular behavior. Understanding this framework requires careful examination of the structural features of Wnt ligands, their cognate Fz receptors, and associated coreceptors, as well as the downstream signaling routes they engage. These pathways, broadly categorized into canonical and noncanonical branches, translate extracellular cues into distinct transcriptional and cytoskeletal outcomes that shape cell fate, polarity, and tissue organization. Moreover, Wnt signaling rarely operates in isolation; instead, it is embedded in a dense network of interactions with other major pathways that collectively fine‐tune physiological and pathological responses. By outlining these structural components, signaling modes, and interpathway connections, this section establishes the mechanistic basis needed to understand how Wnt activity is regulated across diverse biological contexts.

### 2.1 Wnt Ligands, Receptors, and Coreceptors

All three Wnt signaling pathways are initiated by the binding of Wnt ligands to Fz receptors, leading to the intracellular recruitment of dishevelled (Dvl) protein to the Fz complex.

**FIGURE 1:**
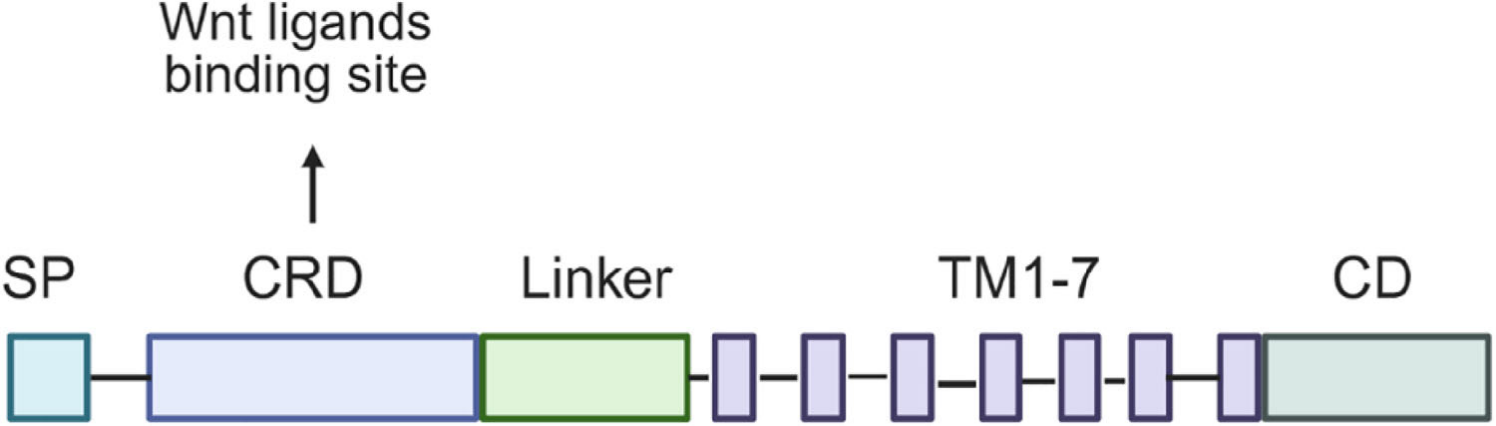
Schematic representation of Fz receptors. SP: signal peptide; CRD: cysteine‐rich domain, responsible for Wnt ligand binding; TM: transmembrane domain; CD: cytoplasmic domain. Created with BioRender.

The Fz receptors belong to the G protein‐coupled receptor family, and their activation requires various coreceptors, such as low‐density lipoprotein receptor‐related protein 5 and 6 (LRP5/6), or the transmembrane (TM) receptor tyrosine kinases ROR1, ROR2, and RYK. Structurally, Fz receptors are characterized by some conserved features (Figure 1), including an N‐terminal signal sequence, a cysteine‐rich domain (CRD) in the extracellular region, a seven TM (7‐TM) domain, and an intracellular C‐terminal domain [35]. The CRD, consisting of approximately 120 amino acids at the N‐terminal, serves as the primary binding site for Wnt ligands. This CRD is linked to the first TM helix by a hydrophilic segment of 70–120 amino acids, which is less conserved among different Fz receptors, contributing to functional diversity [36].

Similarly, the human Fz receptor family consists of 10 members (Fz1 to Fz10), with some displaying pathway‐specific interactions. Fz1 is primarily associated with Wnt/β‐catenin signaling [12], whereas Fz1, Fz2, Fz3, Fz6, and Fz7 preferentially engage in the Wnt/PCP pathway [37]. Some receptors, such as Fz5 and Fz7, can participate in both canonical and noncanonical signaling, depending on ligand availability, coreceptor interactions, and cellular context.

The Wnt ligands are a class of secreted, lipid‐modified glycoproteins, and their glycosylation is essential for secretion. The lipid modification of Wnt ligands involves palmitoylation, which is critical for their binding to Fz receptors. This modification covalently attaches palmitic acid on the first cysteine residue and palmitoleic acid on the highly conserved serine residue to enhance the hydrophobicity of Wnt ligands [13, 35]. Wnt ligands typically form complexes with heparan sulfate proteoglycans to prevent aggregation and unintended activation of Wnt signaling [38].

In humans, the Wnt family comprises 19 members: Wnt1, Wnt2, Wnt2b, Wnt3, Wnt3a, Wnt4, Wnt5a, Wnt5b, Wnt6, Wnt7a, Wnt7b, Wnt8a, Wnt8b, Wnt9a, Wnt9b, Wnt10a, Wnt10b, Wnt11, and Wnt16. While some ligands can activate multiple pathways, others exhibit a preference for specific signaling cascades. For instance, Wnt1, Wnt2, Wnt3, Wnt3a, Wnt8a, Wnt10a, and Wnt10b primarily activate the Wnt/β‐catenin pathway, whereas Wnt5a and Wnt11 are predominantly associated with the Wnt/PCP and Wnt/Ca^2+^ pathways [12, 37, 39, 40, 41]. The signaling preference of certain ligands, such as Wnt4, Wnt5b, and Wnt6, may vary depending on the tissue microenvironment.

### 2.2 The Canonical and Noncanonical Wnt Pathways

**FIGURE 2:**
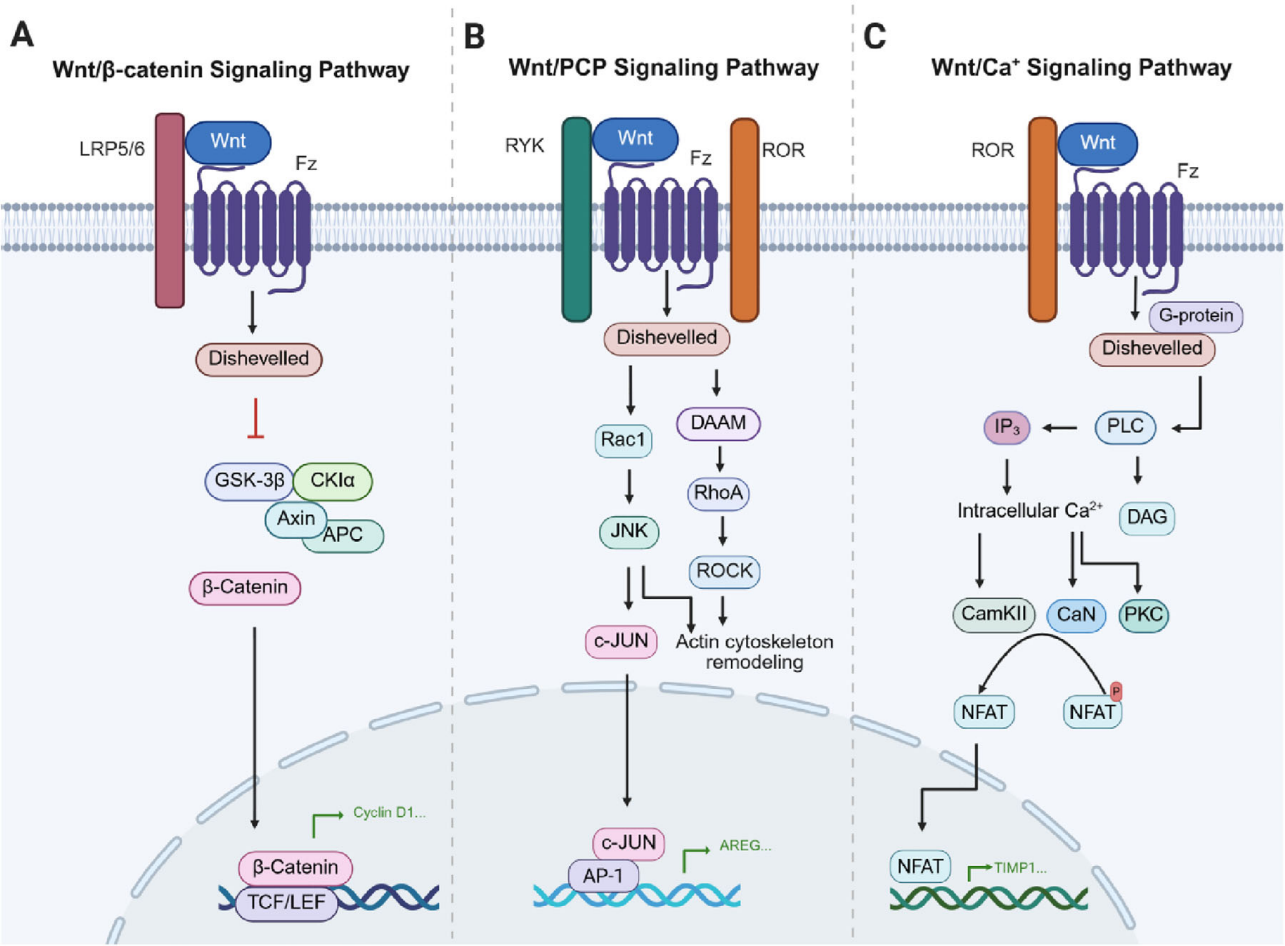
Wnt signaling pathways. (A) Canonical Wnt/β‐catenin pathway: Wnt binding to Fz and LRP receptors disrupts the degradation complex, allowing β‐catenin accumulation and translocation to the nucleus to activate gene transcription. (B) Wnt/PCP pathway: Wnt activation of Dvl leads to RhoA and Rac1 signaling, which regulates JNK activation, cytoskeleton remodeling, and gene transcription. (C) Wnt/Ca^2+^ pathway: Wnt binding activates PLC, increasing cytoplasmic Ca^2^⁺ levels and activating Ca^2^⁺‐sensitive enzymes, which leads to NFAT dephosphorylation and nuclear translocation. Created with BioRender.

The canonical Wnt pathway and noncanonical Wnt pathway differ in their activation patterns and functions. In the canonical pathway (Figure 2A), the absence of Wnt ligands leads to β‐catenin being sequestered in a cytosolic protein complex containing Axin, APC, GSK‐3β, and other proteins. Axin and APC serve as scaffold proteins, facilitating GSK‐3β‐mediated phosphorylation of β‐catenin, which then undergoes ubiquitination and degradation mediated by β‐transducin repeat‐containing homologue protein (β‐TrCP). This process maintains low levels of cytosolic β‐catenin in the absence of ligand stimulation. Upon Wnt ligand binding to Fz receptors and LRP5/6 coreceptors, Dvl is activated, inhibiting GSK‐3β‐mediated β‐catenin phosphorylation. This stabilization allows β‐catenin to accumulate and translocate into the nucleus, where it binds to TCF/LEF family transcription factors, driving the expression of Wnt target genes [14, 42]. Dvl–DEP domain interacting protein (also known as Spats1) acts as a negative regulator of Wnt/β‐catenin signaling by promoting the degradation of TCF and disrupting the formation of the TCF/β‐catenin complex [43].

Unlike the canonical pathway, the Wnt/PCP pathway (Figure 2B) involves the GTP‐dependent activation of small GTPases, such as Rac1 and RhoA. These small GTPases subsequently activate JUN N‐terminal kinase (JNK), ultimately orchestrating cytoskeleton remodeling, cell polarity, and motility [37].

The Wnt/Ca^2+^ pathway (Figure 2C) is initiated when phospholipase C (PLC) interacts with heterotrimeric G proteins. This interaction triggers a signaling cascade that produces diacylglycerol and inositol 1,4,5‐trisphosphate (IP3). The subsequent release of intracellular IP3 elevates calcium ions (Ca^2+^) levels, activating Ca^2+^‐sensitive enzymes like calcium/calmodulin‐dependent protein kinase II (CaMKII) and protein kinase C (PKC). Additionally, calcium‐dependent activation of CaMKII and phosphatase calcineurin (CaN) leads to the dephosphorylation of the nuclear factor of activated T cells, which facilitates its nuclear translocation and the initiation of related genes transcription [44].

### 2.3 Crosstalk With Other Signaling Pathways

**FIGURE 3:**
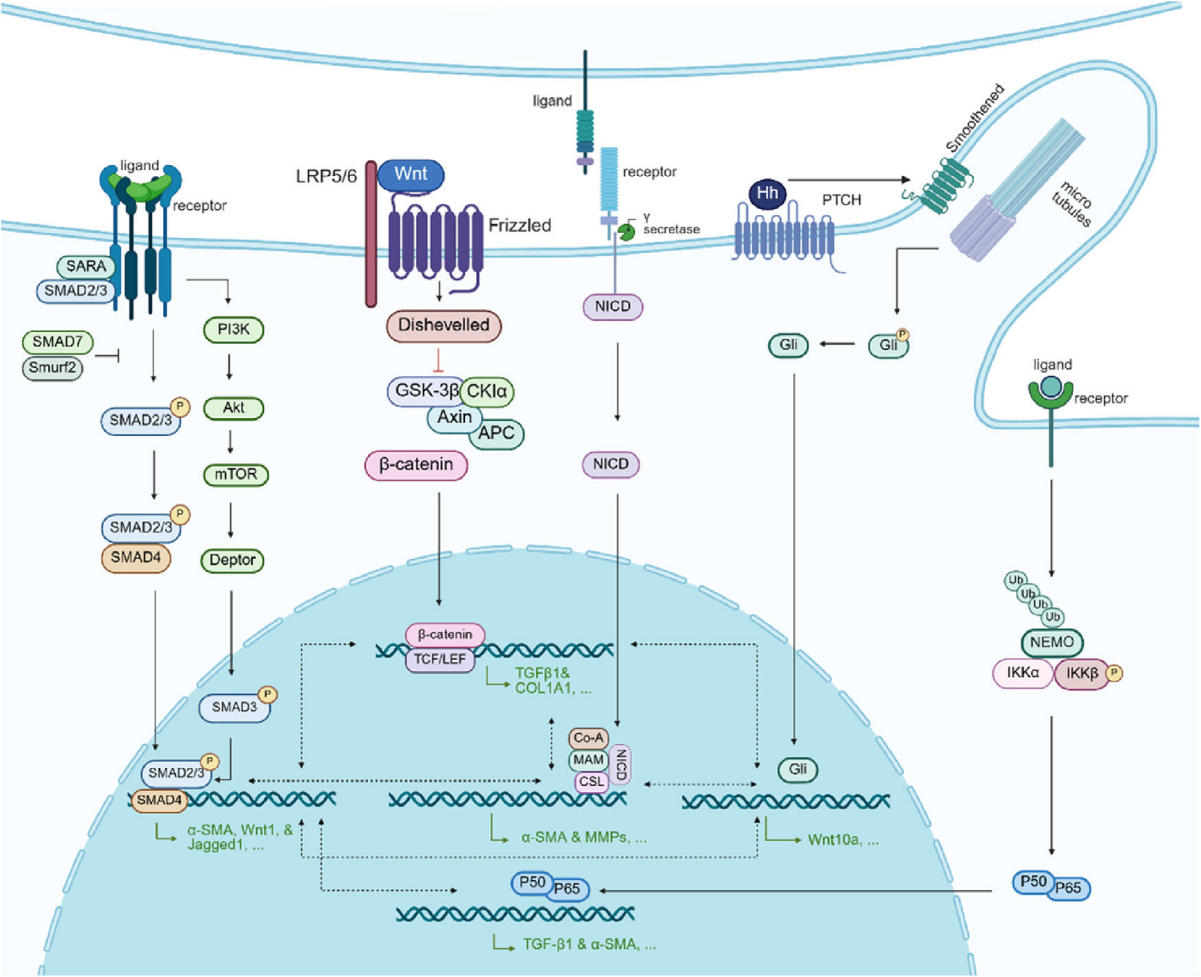
Key signaling pathway networks in tissue fibrosis. The TGF‐β pathway serves as a central regulator of fibrosis, with Wnt signaling enhancing TGF‐β target gene expression and activating Notch signaling to promote fibroblasts differentiation and proliferation. Hedgehog, PI3K/Akt, and NF‐κB pathways further amplify fibrotic responses by driving inflammation, cell proliferation, migration, and ECM synthesis, thereby exacerbating fibrosis progression. Created with BioRender.

The Wnt pathway engages in extensive and intricate crosstalk with multiple signaling networks. In the pathological process of tissue fibrosis, Wnt signaling activates the Notch pathway, further promoting fibrotic cell proliferation and differentiation [45, 46]. Notch, in turn, cooperates with TGF‐β to sustain fibroblasts activation [47, 48, 49]. TGF‐β signaling cascade interacts synergistically with the Wnt/β‐catenin pathway, which exacerbates fibrosis by stabilizing β‐catenin, upregulating fibrotic gene expression, and forming a positive feedback loop with TGF‐β [28, 50, 51, 52, 53, 54]. Additional pathways, such as Hedgehog, PI3K/Akt, and NF‐κB, further modulate inflammation, cell proliferation, migration, and ECM production, collectively shaping the fibrotic response [47, 55, 56, 57, 58, 59, 60, 61, 62, 63, 64, 65, 66]. Together, as illustrated in Figure 3, these pathways constitute a complex and interdependent regulatory network that dictates the onset, progression, and severity of tissue fibrosis.

## 3 Wnt Signaling in Disease and Pathology

The Wnt signaling pathway plays a vital role in governing diverse physiological processes across the lifespan, from embryonic development to adult tissue renewal and homeostasis. Growing evidence from molecular and genetic studies has revealed that perturbations in Wnt activity, either through excessive activation or loss of function, can drive the onset and progression of a wide spectrum of diseases, ranging from developmental malfunctions and cancers to skeletal diseases, neurodegenerative disorders, and fibrotic pathologies, underscoring its broad impact on human health [67, 68, 69, 70, 71].

### 3.1 Developmental Disorders

In normal embryogenesis, Wnt signaling exerts precise control over fundamental biological processes, including cell proliferation, differentiation, and morphogenesis, through spatiotemporal regulation of target gene expression. Both hyperactivation and suppression of this pathway can disrupt developmental programs, resulting in structural anomalies such as neural tube closure (NTC) defects and skeletal malformations [72, 73, 74]. These defects often stem from complex gene–environment interactions, clinically manifest as heterogeneous phenotypes encompassing skeletal abnormalities, congenital malformations, and neurodevelopmental impairments [75].

Down syndrome (DS), the most common chromosomal disorder, exemplifies the critical regulatory role of Wnt signaling during development. Epidemiological data indicate that craniofacial malformations, a hallmark clinical feature affecting approximately 60,000 individuals worldwide, arise primarily from aberrant development of craniofacial bones and soft tissues [76, 77]. Molecular analyses reveal that trisomy 21‐associated gene dosage effects, particularly SOST overexpression and consequent sclerostin (SOST) overproduction, potently inhibit Wnt/β‐catenin signaling. This mechanism not only accounts for characteristic skeletal phenotypes such as brachycephaly and a flattened nasal bridge, but also contributes to cardiovascular malformations and pulmonary hypertension [78]. Of particular note is the biphasic regulatory capacity of DYRK1A: under basal conditions, it suppresses Wnt signaling via phosphorylation cascades impacting neurodevelopment, whereas under activated states, it enhances Wnt activity through protein stabilization mechanisms. Disruption of this delicate equilibrium may contribute to intellectual disability and early‐onset Alzheimer's disease (AD) in DS patients [79].

The pathological consequences of Wnt dysregulation extend beyond DS, serving as a convergent mechanism in multiple genetic disorders. In Klinefelter syndrome, X chromosome aneuploidy and defective X chromosome inactivation result in Wnt pathway overactivation. This aberrant signaling, through synergistic interactions with TGF‐β pathways, impairs fetal germ cell development and differentiation [80]. Similarly, in ciliopathies such as Meckel–Gruber and Joubert syndromes, mutations in ciliary structural proteins (e.g., MKS1) impair primary cilium‐mediated signal transduction, leading to canonical Wnt/β‐catenin overactivation. These defects compromise cilia‐dependent morphogenetic processes, generating complex malformation spectra including polycystic kidneys and polydactyly [81]. Collectively, these examples underscore that precise spatiotemporal control of Wnt signaling is essential for proper organogenesis, and its dysregulation precipitate diverse congenital abnormalities.

Neural tube defects, resulting from failed neural tube closure (NTC), rank among the most severe human developmental disorders [82]. Wnt signaling coordinates NTC through both canonical and noncanonical pathways. In the canonical Wnt/β‐catenin pathway, ligand binding to Fz and LRP5/6 receptors stabilizes β‐catenin by inhibiting its degradation via the Axin/APC/GSK‐3β/CK1 complex. Stabilized β‐catenin translocates to the nucleus, where it interacts with TCF/LEF transcription factors to regulate key developmental genes such as Axin2 and Msx1 [67, 83, 84]. Experimental evidence demonstrate that folate deficiency triggers abnormal Wnt/β‐catenin activation via Gcm1 upregulation, which disrupts Axin2 expression and creates an imbalance between proliferation and apoptosis in neuroepithelial cells, ultimately compromising the cellular foundation required for proper NTC [85]. Conversely, the noncanonical Wnt/PCP pathway governs NTC by directing cell polarity and convergent extension movements during neural plate morphogenesis [86].

### 3.2 Cancer Pathogenesis

The Wnt signaling cascade is a conserved regulator of cellular processes, and its dysregulation is a key driver of oncogenesis. Aberrant activation contributes to tumor initiation, progression, and metastasis through mechanisms including genomic alterations, epigenetic modifications, and microenvironmental interactions [69]. Moreover, Wnt signaling sustains cancer stem cells properties, supporting self‐renewal, therapy resistance, and metastatic potential.

Canonical Wnt/β‐catenin signaling is frequently hyperactivated in cancers, often via mutations in pathway components such as APC or β‐catenin. In colorectal cancer, these mutations stabilize β‐catenin, enabling TCF/LEF‐mediated transcription of oncogenes like MYC and matrix metalloproteinases (MMP)9 [87, 88]. Parallel mechanisms in esophageal squamous cell carcinoma, cholangiocarcinoma, and hepatocellular carcinoma highlight tumor‐specific pathway activation, while stromal cells in the tumor microenvironment can further amplify Wnt signaling via paracrine ligand secretion, exemplifying multicellular regulatory networks [89, 90, 91, 92, 93, 94].

Wnt signaling also maintains CSC traits across cancer types. In colorectal and hepatocellular carcinoma, β‐catenin stabilization promotes stemness and therapy resistance, while noncanonical pathways, such as Wnt5a–ROR2, regulate vascular remodeling and invasion in melanoma, breast cancer, and glioblastoma [95]. Epigenetic regulators, including lncRNAs and m6A‐modified transcripts, modulate these processes, providing additional layers of pathway control [96, 97, 98, 99, 100, 101, 102].

During metastasis, Wnt signaling orchestrates context‐specific mechanisms. EMT induction via ligand–receptor axes (e.g., Wnt3a–FZD7) facilitates initial dissemination [92, 103], while microenvironmental cues, including Wnt5a‐mediated vascular remodeling and immunosuppressive niche formation, support metastatic colonization. Notably, some ligands exhibit dual roles, promoting metastasis through noncanonical targets while suppressing canonical Wnt signaling, underscoring the complexity of therapeutic targeting [100, 102, 104, 105, 106, 107].

### 3.3 Skeletal System Pathologies

Osteoporosis, osteoarthritis (OA), and impaired fracture healing represent major skeletal disorders in which Wnt signaling is a central regulator of bone formation, remodeling, and morphogenesis. Canonical Wnt/β‐catenin signaling promotes osteoblast differentiation and osteogenic gene expression, while noncanonical Wnt/PCP and Wnt/Ca^2+^ pathways coordinate skeletal morphogenesis, cell polarity, and metabolic balance [108]. Dysregulation of these pathways contributes to the pathophysiology of diverse bone diseases.

In osteoporosis, Wnt/β‐catenin signaling promotes Runx2 and Osterix expression, enhancing osteogenesis [108]. SOST inhibits Wnt signaling by competing for LRP5/6 binding, suppressing bone formation [109]. SOST‐targeting aptamers like Apc001OA restore Wnt activity, enhance osteogenic differentiation, and promote matrix mineralization [110]. Noncanonical Wnt5a–ROR2 signaling bifurcates into Ca^2+^/CaMKII‐mediated osteoclast precursor migration and JNK/c‐Jun‐mediated suppression of osteoblast activity, maintaining a balance between bone resorption and formation [111]. Genetic variants in Wnt1, Wnt5b, and ARHGEF15 further influence skeletal homeostasis by altering ligand expression, secretion, and cytoskeletal signaling [112, 113].

In OA, moderate canonical Wnt activity preserves chondrocyte homeostasis, whereas excessive activation causes β‐catenin accumulation, chondrocyte hypertrophy, and upregulation of matrix‐degrading enzymes (MMP13, ADAMTS), accelerating cartilage degeneration [114, 115]. Correspondingly, loss of Wnt antagonists such as SOST or Dickkopf (DKK)1 exacerbates disease progression [116, 117]. Noncanonical Wnt5a–ROR2 signaling promotes inflammation and cartilage breakdown, whereas via PCP/JNK–mTORC1–PTHrP signaling preserves chondrocyte integrity [118, 119, 120]. Crosstalk with bone morphogenetic protein (BMP) and PTH signaling further fine‐tunes bone‐cartilage metabolic balance, illustrating the multidimensional regulation of skeletal tissue [116, 121].

Fracture healing relies on spatiotemporally coordinated Wnt signaling. Early canonical Wnt3a activation drives chondrocyte‐to‐osteoblast conversion, callus mineralization, and periosteal stem cell proliferation [14, 122]. Noncanonical Wnt5a–RhoA/ROCK signaling regulates cellular polarity, migration, inflammatory cell infiltration, and angiogenesis [111, 123]. Coordinated interplay between canonical and noncanonical pathways ensures orderly tissue regeneration, while modulators such as SOST and DKK1 can delay healing, and activators like WISP‐1 enhance cartilage proliferation [108, 124].

### 3.4 Neurodegenerative Diseases

Neurodegenerative disorders such as AD, Parkinson's disease (PD), and Huntington's disease (HD) involve progressive neuronal loss and synaptic dysfunction. Wnt signaling supports neuronal survival, synaptic plasticity, and mitochondrial integrity, while its dysregulation contributes to disease onset and progression [125, 126, 127].

In AD, canonical Wnt/β‐catenin signaling inhibits amyloid‐β (Aβ) production, reduces tau hyperphosphorylation, and maintains synaptic stability [128]. Aβ disrupts this pathway by competing for FZD binding and activating GSK‐3β, creating a feedback loop that accelerates pathology [129, 130, 131, 132]. Noncanonical pathways, such as Wnt5a, influence mitochondrial function and neuroinflammation but may also promote synaptic loss [133, 134, 135]. Restoring Wnt activity via MST1 inhibition or genetic upregulation of Wnt2a/APOE3 Christchurch improves synaptic function and reduces protein aggregates in models [136, 137, 138, 139].

In PD, Wnt signaling regulates dopaminergic neuron differentiation, survival, and glial–neuronal communication [140, 141, 142]. Canonical (Wnt1/3a) and noncanonical (Wnt5a) pathways act synergistically, with β‐catenin stabilization and GSK‐3β inhibition conferring neuroprotection [143, 144, 145]. Mutations such as LRRK2 G2019S impair Wnt signaling, whereas Wnt1 activation promotes neurogenesis and functional recovery [146, 147, 148, 149].

In HD, mutant huntingtin binds β‐catenin, preventing its degradation and altering gene transcription [150]. This overactivation, along with GSK‐3β downregulation, creates a proapoptotic environment [151]. While excessive Wnt signaling drives abnormal neural progenitor proliferation [152, 153], moderate activation can protect neurons [154], indicating a biphasic, context‐dependent role.

### 3.5 Fibrotic Disorders

Fibrosis is a conserved pathological response to chronic injury, marked by excessive ECM deposition that disrupts tissue architecture and function. It occurs across diverse contexts, including metabolic, vascular, and autoimmune disorders such as systemic sclerosis (SSc), and follows a common injury–inflammation–fibrosis axis despite organ‐specific triggers [155].

Fibrotic progression typically initiates with epithelial injury, triggering profibrotic factor release (e.g., TGF‐β, IL‐1α) that activates fibroblasts and drives myofibroblast differentiation, expressing α‐smooth muscle actin (α‐SMA) [156, 157]. They are the primary effector cells of fibrosis, secreting fibrillar collagens (types I and III) and remodeling the matrix to create tissue stiffening and architectural distortion [155]. Concurrently, the injury site becomes infiltrated by various immune cell populations, most notably macrophages and T lymphocytes, which establish a proinflammatory microenvironment through sustained secretion of both inflammatory cytokines and additional fibrogenic factors [158]. Immune cell infiltration, particularly by macrophages and T cells, sustains inflammation and amplifies fibrogenic signaling. ECM stiffening further reinforces myofibroblast activation through mechanotransduction pathways such as integrins, YAP/TAZ, and MRTF, while imbalance between MMPs and their inhibitors impairs ECM turnover, locking the system into a self‐perpetuating fibrotic state [159]. These interconnected molecular and cellular events collectively constitute a conserved “injury–inflammation–fibrosis” axis that operates across diverse organ systems, creating a self‐perpetuating cycle of fibrogenesis that underlies the progression of chronic fibrotic diseases.

Wnt signaling modulates fibrosis through multiple organ‐specific mechanisms. In renal fibrosis, canonical Wnt/β‐catenin signaling promotes endothelial–mesenchymal transition (EndMT) [160], while noncanonical Wnt5a enhances TGF‐β1‐induced YAP/TAZ expression, promoting macrophage M2 polarization and fibrosis exacerbation [161]. Pulmonary fibrosis involves Wnt4‐mediated alveolar type 2 (AT2) cell proliferation via YAP/TAZ [162], while persistent IL‐1β signaling overactivates Wnt/β‐catenin pathways, impairing AT2‐to‐AT1 differentiation and causing abnormal transitional cell accumulation [163]. Liver fibrosis progression involves R‐spondin (RSPO)1/2/3–LGR4/5 axis‐mediated Wnt receptor stabilization and hepatic stellate cell (HSC) activation, coupled with glycolytic reprogramming [68]. Skin fibrosis demonstrates Wnt/β‐catenin‐TGF‐β synergy promoting myofibroblast transformation and ECM deposition, counterbalanced by IL‐1β‐mediated AKT phosphorylation inhibition and β‐catenin degradation [164]. While regulatory molecules vary, core mechanisms consistently involve cellular phenotype modulation, inflammatory regulation, and ECM metabolic imbalance, suggesting Wnt pathway modulation as a potential pan‐fibrotic therapeutic strategy requiring organ‐specific optimization to minimize off‐target effects.

## 4 Regulation of Wnt Signaling in Tissue Fibrosis

Regulation of Wnt signaling in tissue fibrosis involves a multilayered network of modulators that collectively determine the amplitude, duration, and specificity of Wnt pathway activity. These regulatory mechanisms do not simply turn signaling “on” or “off”; rather, they fine‐tune ligand availability, receptor stability, and downstream signal propagation in ways that shape fibroblast activation, matrix deposition, and tissue remodeling. Negative regulators such as DKKs, Wnt inhibitory factor‐1 (WIF‐1), IGF‐binding protein 4 (IGFBP4), ZNRF3/RNF43, and Notum impose critical constraints on pathway activation, while positive modulators, including ROR/RYK coreceptors, the RSPO–LGR axis, and phosphatases such as protein phosphatase 2A (PP2A), potentiate canonical or noncanonical Wnt responses depending on cellular context. Added to this regulatory landscape are the SFRP family members, which embody the context‐dependency of Wnt modulation by functioning as inhibitors, stabilizers, or even facilitators of Wnt signaling. Together, these diverse regulatory inputs outline a dynamic control system that governs how Wnt signaling contributes to the initiation, progression, or resolution of fibrosis across different tissues.

### 4.1 Negative Regulators

#### 4.1.1 DKK Family

The DKK family, comprising DKK1, DKK2, DKK3, and DKK4, modulates Wnt signaling by binding to the LRP6 coreceptor and inhibiting β‐catenin‐dependent pathways. DKK1, DKK2, and DKK4 share high sequence homology and generally exhibit antifibrotic effects. In contrast, DKK3, due to structural differences and inability to mediate LRP6 internalization via Kremen receptors, demonstrates context‐dependent functions, often with profibrotic roles [165, 166].

In high glucose‐induced diabetic mouse models, DKK1 inhibition reduces TGF‐β1 and fibronectin expression, decreases glomerular hypertrophy, and limits mesangial matrix expansion by destabilizing β‐catenin [167]. DKK1 also suppresses pericyte activation and myofibroblast proliferation after kidney injury by disrupting TGF‐β and connective tissue growth factor (CTGF)‐mediated MAPK/JNK signaling via LRP6 interaction [168]. Downregulation of KLF10 ameliorates diabetic renal fibrosis through reduced DKK1, TGF‐β1, and phosphorylated β‐catenin expression [169]. Unlike DKK1 and DKK2, DKK3 is strongly associated with renal fibrosis progression, with urinary levels correlating with tubular atrophy and interstitial fibrosis in chronic kidney disease [170].

#### 4.1.2 Wnt Inhibitory Factor

WIF‐1 antagonizes Wnt signaling by directly binding to Wnt ligands, preventing receptor engagement [14]. In SSc, WIF‐1 is downregulated in fibroblasts, accompanied by elevated Wnt activity and collagen accumulation. This repression is driven by oxidative DNA damage that recruits c‐Jun, ATF3, and HDAC3 to the WIF‐1 promoter [171]. In pulmonary fibrosis, WIF‐1 downregulation, partly due to promoter hypermethylation, can be reversed by knockdown of its upstream repressor MeCP2 [172]. Notably, in thioacetamide‐induced liver fibrosis model, treatment with dasatinib restores WIF‐1 expression via miR‐17 downregulation, suppressing Wnt/β‐catenin and downstream TGFβ/Smad pathways [173].

#### 4.1.3 Others

IGFBP4 antagonizes Wnt/β‐catenin signaling and is implicated in fibrosis regulation across tissues. Reduced IGFBP4 levels correlate with steatosis severity in NAFLD [174] and are observed in Crohn's disease and peritoneal fibrosis, where supplementation can suppress TGF‐β1‐induced mesothelial‐to‐mesenchymal transition [175, 176]. IGFBP4 also limits ECM production in SSc‐associated lung and skin fibrosis [177].

ZNRF3 and RNF43 are TM E3 ubiquitin ligases that degrade β‐catenin in the absence of Wnt ligands [68, 178]. Hepatocyte‐specific loss of these proteins leads to NASH‐like changes and disrupts metabolic liver zonation [179, 180, 181].

Notum suppresses canonical Wnt signaling by deacylating Wnt ligands. In HBV‐induced liver fibrosis, Notum downregulation of Wnt5a signaling exerts antifibrotic effects [182]. AAV‐mediated Notum overexpression in obese mice improves adipose thermogenesis and reduces fibrosis [183].

### 4.2 Positive Modulators

#### 4.2.1 ROR/RYK

Receptor tyrosine kinase‐like orphan receptors (ROR1/2) and related tyrosine kinase (RYK) act as Wnt‐binding receptors [184]. ROR1/2 predominantly mediate Wnt5a‐driven noncanonical signaling [185].

In cardiac fibrosis, ROR1/2 expression is elevated during fibroblast activation and ECM deposition, while ROR1/2 deficiency reduces fibrosis [186]. Similarly, Wnt5a–ROR signaling is crucial in IPF [187, 188], with Wnt5a loss‐of‐function showed markedly reduced alveologenesis and myofibroblast migration in mice [189]. Beyond the lung, ROR1 serves as a circulating biomarker in kidney fibrosis [190] and contributes to subretinal fibrosis in neovascular AMD [191]. In peritoneal fibrosis, the role of Wnt5a is context dependent: its profibrotic or antifibrotic effects depends on ROR2 expression, with ROR2 silencing reversing the antifibrotic and antiangiogenic activity of Wnt5a [192].

RYK shows a dual role depending on tissue context. In the nervous system, RYK is upregulated after nerve injury and spinal cord trauma, and astrocyte‐specific RYK deletion accelerates scar border formation and functional recovery [193, 194]. Conversely, in the liver, elevated RYK expression in activated HSCs supports fibrosis progression [195].

#### 4.2.2 RSPO and LGR4/5

The RSPO family, acting through their receptors LGR4 and LGR5, primarily enhances Wnt signaling by promoting degradation of the E3 ligases ZNRF3 and RNF43, thereby stabilizing Fz receptors. Although RSPOs are widely implicated in fibrosis, their roles vary across tissues and even among family members.

RSPO1 is generally profibrotic. It is overexpressed in fibrotic human livers and culture‐activated HSCs, driving fibrogenesis via canonical Wnt signaling, which can be reversed by the antagonist DKK1 [196]. In obesity‐associated renal fibrosis, RSPO1 is elevated in high‐fat diet mice, promoting kidney dysfunction and fibrosis; LGR4 knockdown alleviates these effects [197]. Similarly, LGR4 knockout mice develop polycystic kidney lesions and fibrosis via Wnt‐dependent but TGF‐β/Smad‐independent mechanisms [198].

In contrast, RSPO3 exerts antifibrotic functions in both the liver and pancreas. In the liver, RSPO3 expression declines during HSC activation and correlates inversely with fibrosis severity in alcohol‐related and metabolic steatotic liver disease (MASLD) [199, 200]. HSC‐derived RSPO3 maintains liver homeostasis, regulates zonation and metabolism, and prevents fibrosis. In MASLD, RSPO3 protects against steatosis, injury, and fibrosis by inhibiting HSC activation [199]. In the pancreas, RSPO3 is enriched in Meflin^+^ stellate cells after injury and appears to limit fibrosis progression [201].

RSPO2 shows context‐dependent roles. In keloids, keratinocytes stimulate fibroblasts to secrete RSPO2, promoting keratinocyte proliferation and epidermal thickening [202]. Conversely, in idiopathic pulmonary fibrosis (IPF), RSPO2 and its receptor LGR6 are upregulated in fibroblasts and epithelial cells. RSPO2 stimulation inhibits fibroblast proliferation and collagen deposition while promoting apoptosis via Wnt signaling, thereby exerting antifibrotic effects [203].

The receptors themselves also display dual functions. In skin, LGR5 expressed in a fibroblast subset termed scleroderma‐associated fibroblasts, is dysregulated in SSc, implicating it as a potential therapeutic target [204]. In the liver, LGR5 knockdown worsens carbon tetrachloride (CCl_4_)‐induced fibrosis, whereas administration of HGF with RSPO1 expands LGR5^+^ liver stem cells and improves function [205]. Growth differentiation factor 11, elevated in fibrosis, further supports antifibrotic repair partly by expanding LGR5^+^ progenitors [206]. In contrast, LGR4 shows a more profibrotic profile: hepatocyte‐specific LGR4 depletion attenuates bile duct ligation‐induced injury and fibrosis [207], and activated LGR4‐β‐catenin signaling contributes to miR‐122 overexpression‐induced cardiovascular fibrosis [208].

#### 4.2.3 Protein Phosphatase 2A

PP2A regulates Wnt signaling by dephosphorylating β‐catenin, thereby stabilizing it in the cytoplasm and enhancing signal transduction. PP2A has been implicated in the fibrotic processes across several organs.

In the kidney, PP2A promotes fibroblast activation and fibrosis, by inhibiting the ERK pathway [209], or by increasing AMP‐activated protein kinase (AMPK) dephosphorylation, leading to impaired fatty acid oxidation [210]. PP2A plays a dual role in liver fibrogenesis. On one hand, KISS1R suppresses TGF‐β signaling and attenuates fibrosis in MASLD through PP2A‐mediated SMAD2/3 dephosphorylation [211]. On the other hand, hepatocyte‐specific deletion of PP2A Aα subunit causes spontaneous fibrosis in mice [212]. Elevated PP2Acα is also observed in CCl_4_‐induced liver injury and in patients with subacute hepatitis, where its inhibition reduces fibrosis via the ASK/JNK pathway [213]. In the heart, the PP2A/HDAC2 axis regulates hypertrophic remodeling. PPP2CA overexpression protects against isoproterenol‐induced hypertrophy and fibrosis [214]. However, excessive myocardial PP2A overexpression aggravates post‐MI hypertrophy and fibrosis [215].

### 4.3 SFRPs: Context‐Dependent Wnt Modulators

#### 4.3.1 Structural Domains and Binding Specificity of SFRPs

**FIGURE 4:**
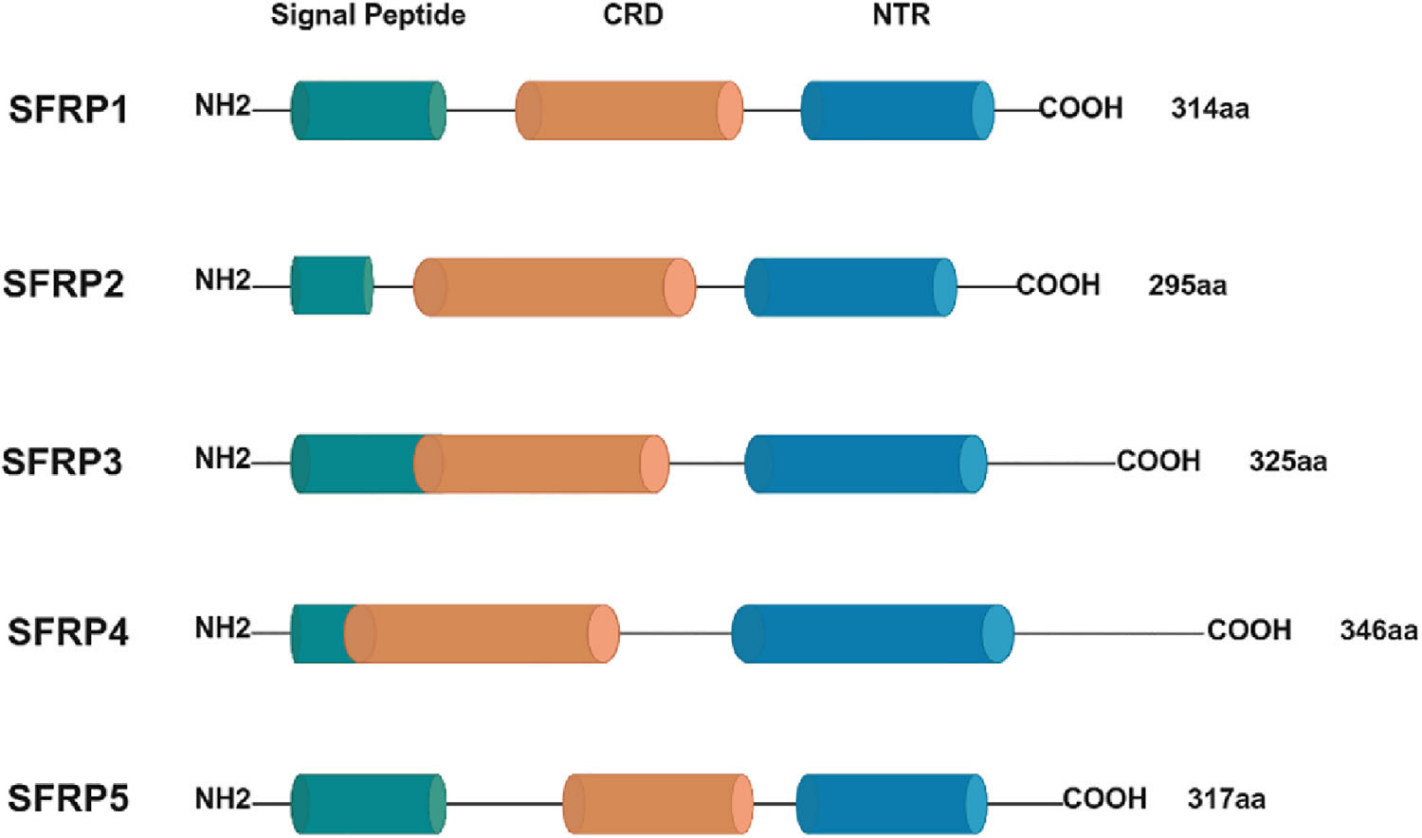
Structure of the SFRP family members. The SFRPs are composed of three main domains: the signal peptide (green), CRD (orange), and NTR (blue). This figure is modified from Zhang et al. [216]. Created with BioRender.

As illustrated in Figure 4, SFRPs consist of three key domains: a N‐terminal signal peptide, a coiled CRD, and a C‐terminal netrin‐like domain (NTR) [216, 217]. The CRD, approximately 120 amino acids long, contains 10 conserved cysteine residues and shares 30–50% sequence similarity with the Wnt‐binding region of Fz receptors [218, 219]. The NTR domain includes six cysteine residues and several conserved hydrophobic residues [220], whereas the signal peptide plays a role in the secretion and maturation of SFRPs [221].

#### 4.3.2 Dual Roles of SFRPs in Wnt Signaling Regulation

**FIGURE 5:**
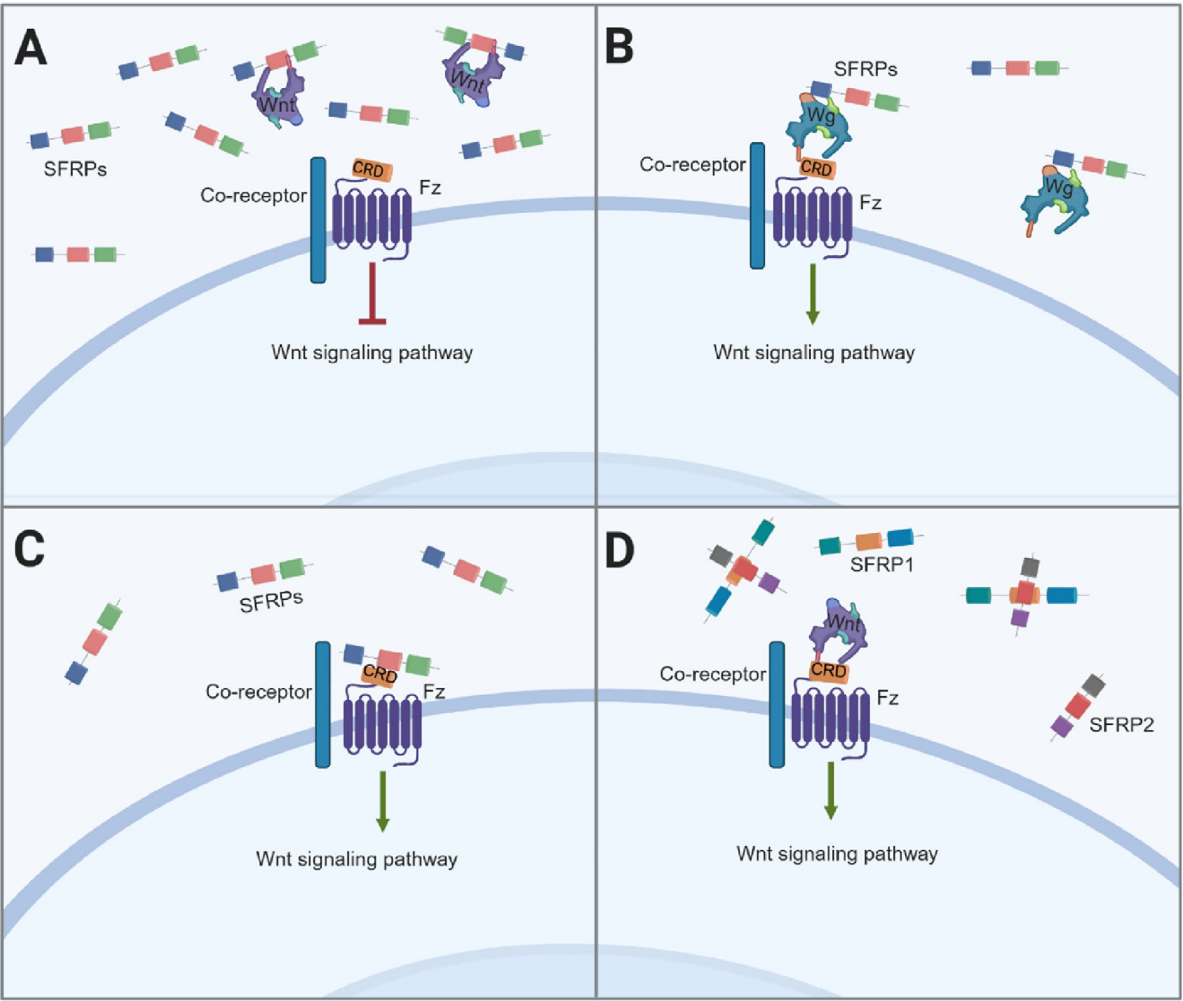
Diverse mechanisms of SFRP‐mediated modulation of Wnt signaling. (A) SFRPs act as classical antagonists by sequestering Wnt ligands through their CRD domain, preventing Wnt–Fz interaction. (B) SFRPs stabilize Wnt ligands by binding to high‐affinity sites, enhancing their recognition by Fz receptors and promoting signal transduction. (C) SFRPs interact directly with Fz receptors, functioning as ligands to activate Wnt signaling. (D) Different SFRP family members antagonize each other's activity, indirectly promoting Wnt signal transduction. Created with BioRender.

Traditionally, SFRPs were considered antagonists due to their structurally similar CRD to Fz receptors, which allows them to compete for Wnt ligand binding at low‐affinity sites. This competition disrupts interaction between Wnt ligands and Fz receptors, effectively inhibiting downstream signaling [222, 223, 224], as depicted in Figure 5A. For example, SFRP1 and SFRP2 suppress Wnt3a‐induced Wnt/β‐catenin and play a role in dorsal neural tube development [225].

However, recent findings reveal that SFRPs can also promote Wnt signaling. Instead of solely binding Wnt at low‐affinity sites, SFRPs at low concentrations can interact with high‐affinity sites via their C‐terminal domain, stabilizing Wnt ligands and enhancing signal transduction (Figure 5B). For instance, low concentrations of SFRP1 stabilize Wg, the *Drosophila* homolog of Wnt, preventing its degradation and increasing levels of Arm, the β‐catenin equivalent in *Drosophila* [34].

Moreover, biochemical and crystallographic studies indicate that the CRD of SFRPs can form homodimers or heterodimers with Fz CRDs, effectively functioning as ligands (Figure 5C). In vertebrates, SFRP1 is highly expressed in key visual pathway regions and serves as a Wnt‐independent axon guidance cue for retinal ganglion cell (RGC) growth cones. Direct interaction between SFRP1 and Fz2 in RGCs mediates this effect, and interfering with Fz2 disrupts SFRP1‐induced axonal responses, highlighting its role as an active ligand in Fz‐mediated signaling [32, 226].

Beyond their interactions with Wnt and Fz, SFRPs also modulate signaling through intrafamily interactions. CRDs of different SFRPs can bind to one another, leading to antagonistic effects (Figure 5D). In embryonic rat kidneys, SFRP1 inhibits Wnt4‐dependent tubule formation and branching morphogenesis. While SFRP2 alone has no effect, its coexpression with SFRP1 counteracts SFRP1's inhibition, partially restoring tubule differentiation and branching [227, 228].

In addition to these mechanistic modes of action, several molecular determinants dictate whether an SFRP functions as a Wnt agonist or antagonist in a given context. Concentration is one of the most critical factors: low SFRP levels favor ligand stabilization or Fz sensitization, whereas high concentrations typically promote competitive inhibition of Wnt–Fz interactions. This principle is reflected in tissue fibrosis. For example, recombinant Sfrp2 at high doses suppresses collagen synthesis and alleviates cardiac fibrosis, while lower levels enhance Wnt signaling in other tissue environments [229, 230]. Tissue‐specific expression patterns of Wnt ligands, Fz receptors, and coreceptors further modulate SFRP activity, as do structural features such as CRD‐mediated dimerization or C‐terminal domain accessibility, which influence binding affinity and receptor engagement [231]. Posttranslational modifications, including glycosylation and proteolytic processing reported for SFRP1 and SFRP2, may additionally alter protein stability, subcellular localization, or receptor preference, thereby shifting functional output [232]. Epigenetic regulation constitutes another important layer, as hypermethylation‐driven downregulation of SFRP1 or SFRP5 in fibrotic tissues (e.g., skin, lung, kidney, and SSc) reduces their antifibrotic potential and skews Wnt pathway activity [233, 234, 235, 236, 237, 238, 239]. Finally, the relative abundance of different SFRP family members, such as the antagonistic SFRP1–SFRP2 interactions described above [227, 228], can redefine pathway dynamics within a given microenvironment. Collectively, these variables underscore that SFRPs do not act through a fixed inhibitory or activating logic but rather operate as context‐dependent modulators, with their functional polarity emerging from the interplay of concentration, tissue milieu, biochemical modifications, and intrafamily dynamics.

## 5 Roles of SFRPs in Tissue Fibrosis: Organ‐Specific Insights

**FIGURE 6:**
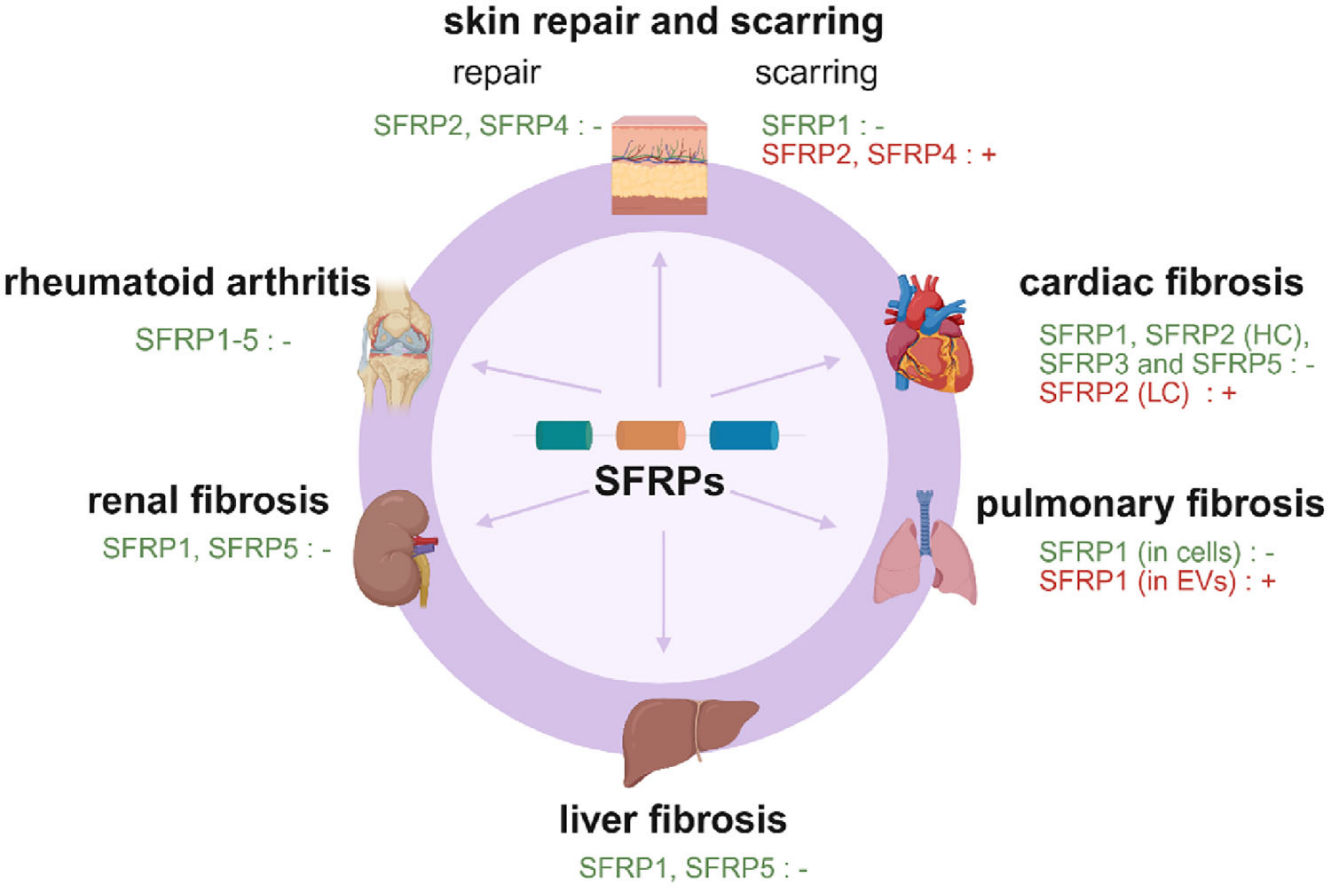
Functions of SFRPs in tissue repair and fibrosis across different organs. SFRPs exhibit diverse effects on fibrosis in skin, heart, lung, liver, kidney, and joint. HC, high concentration; LC, low concentration. EVs, extracellular vesicles. Green texts and “‐” indicate beneficial effects, while red texts and “+” indicate detrimental effects. Created with BioRender.

Unlike WIF1, SFRPs regulate Wnt signaling through both inhibitory and activating mechanisms. This dual functionality is particularly evident in tissue fibrosis, where different SFRP family members exert distinct regulatory effects. For example, SFRP1, which is broadly expressed across tissues, generally mitigates fibrosis. In contrast, SFRP2 exhibits tissue‐specific roles, acting as an antifibrotic factor in rheumatoid arthritis (RA) while promoting fibrosis by enhancing collagen synthesis in human hypertrophic scars. Notably, epigenetic mechanisms, including DNA hypermethylation and histone modifications, constitute a common regulatory layer controlling SFRP expression across multiple fibrotic diseases. Reduced expression of SFRP1 and SFRP5 caused by promoter hypermethylation has been documented in skin, oral submucosa, lung, kidney fibrosis, and SSc, while demethylating agents can partially reverse these fibrotic phenotypes. The following sections outline the roles of SFRP1–SFRP5 in fibrosis across various organs and tissues (Figure 6). A summary of SFRP family members’ roles in different fibrosis conditions is provided in Table 1.

**TABLE 1:** Roles of SFRPs in fibrosis across different organs and tissues.

Tissues	Cell types	SFRPs	Species	Pathways	Function	References
Skin	Wound fibroblasts	SFRP2	H*	Unclear	Prohealing	[240, 241, 242, 243]
	Scar fibroblasts	SFRP1	H	Wnt/β‐catenin	Antifibrosis	[233, 244]
		SFRP2	H	Unclear	Profibrosis	[245, 246, 247, 248]
		SFRP4	H	Hippo/YAP‐TAZ		[249]
	Palmar fascia fibroblasts	SFRP4	H	NF‐κB/STAT3		[250, 251]
	Oral mucosal fibroblasts	SFRP1	H	Wnt/β‐catenin	Antifibrosis	[252, 253]
		SFRP5	H			[252]
Heart	Cardiac fibroblasts (CFs)	SFRP1	R*	Wnt/β‐catenin	Antifibrosis	[254]
		SFRP2 (HC)	R	Unclear		[229]
		SFRP5	H	Unclear		[255, 256]
	Cardiomyocytes	SFRP1	M	Wnt/β‐catenin		[257]
		SFRP2	R	Wnt/β‐catenin		[230, 258, 259]
		SFRP3	M, R	Unclear		[260]
		SFRP4	R	Wnt/β‐catenin		[261]
		SFRP5	R	AMPK		[262]
	Mesenchymal stem cells (MSCs)	SFRP2	M, R	Wnt/β‐catenin		[258, 263]
	Endothelial cells	SFRP2	H	Unclear		[230]
		SFRP5	H	Wnt5a/JNK		[264]
		SFRP3	Sheep	Wnt/β‐catenin		[265]
	CFs	SFRP2 (LC)	M	Wnt/β‐catenin TNAP and GAPDH	Profibrosis	[266]
	Cardiomyocytes	SFRP4	M	PI3K/AKT		[267]
Lung	Pulmonary fibroblasts	SFRP1	M, H*	RhoA, Wnt/β‐catenin	Antifibrosis	[237, 268]
		SFRP4	M	Wnt/β‐catenin		[237]
	Bronchoalveolar stem cells (BASCs)	SFRP1	M	Unclear		[269]
	Small airway epithelial cells (SAECs)		M	Unclear		[269]
	Extracellular vesicles (EVs)		M	Unclear	Profibrosis	[270]
Liver	Hepatic stellate cells (HSCs)	SFRP1	M, H	Wnt/β‐catenin	Antifibrosis	[271, 272]
		SFRP5	M	Wnt5a/JNK		[273]
Kidney	Renal tubular epithelial cells	SFRP1	M, R	Wnt/β‐catenin, Wnt/PCP		[274, 275]
		SFRP5	M, R	Wnt/β‐catenin		[238, 276]
Inflamed tissue	Dermal fibroblast	SFRP1	H*	Wnt/β‐catenin	Antifibrosis	[239]
		SFRP2	H	Unclear	Profibrosis	[277, 278]
		SFRP4	H	Unclear		[277]
	Synovial fibroblast	SFRP1	H	Wnt/β‐catenin	Antifibrosis	[279]
		SFRP2	R			[280, 281]
		SFRP3	H			[282]
		SFRP4	R			[283]
		SFRP5	H	JNK		[284]

Abbreviations: H, human; HC, high concentration; LC, low concentration; M, mice.

### 5.1 SFRPs in Skin Wound Healing and Scarring

Research on SFRPs in skin wound healing and scarring has primarily focused on SFRP1, SFRP2, and SFRP4, revealing distinct and sometimes opposing roles in fibrosis and repair.

SFRP2 promotes both wound healing and scar formation. In diabetic foot ulcers (DFUs), SFRP2 is highly upregulated in a subset of CD26^+^ fibroblasts, which are enriched in healing DFUs compared with nonhealers. These CD26^+^SFRP2^+^ cells, characterized by a thin tubular morphology, exhibit high expression of ECM‐related genes such as MMP1 and MMP3, supporting ECM remodeling and repair [240, 241, 242]. Additionally, SFRP2 secreted by these fibroblasts facilitates intercellular communication, further enhancing wound healing [243].

In the context of skin scarring, SFRP2 is elevated in fibrotic skin conditions, including hypertrophic scars and scleroderma‐like skin, where it contributes to fibroblasts proliferation and myofibroblasts differentiation. Knockdown of SFRP2 via siRNA suppresses these processes [245]. Mechanistically, SFRP2 promotes the expression of transcription factor Slug, which inhibits fibroblasts apoptosis by regulating Bcl‐2, Bax, and Puma, thereby sustaining excessive fibroblasts activity and scar formation [246, 247, 285]. Additionally, SFRP2 plays a key role in collagen maturation, working with procollagen C‐proteinase enhancer 1 (PCPE1) to regulate BMP1‐mediated procollagen processing. Loss of SFRP2 or PCPE1 impairs collagen I maturation, as seen in SFRP2/PCPE1‐deficient fibroblasts and zebrafish models, further highlighting its involvement in fibrosis [248].

Similarly, SFRP4 has been shown as a driver of fibrosis in hidradenitis suppurativa (HS) and Dupuytren's disease (DD), both characterized by chronic fibrosis. In HS, SFRP4^+^ fibroblasts are disproportionately enriched in lesions, expressing Hippo pathway components (YAP, TAZ, TEAD), which drive their transition into myofibroblasts, leading to excessive collagen deposition and fibrosis [249]. Inhibition of the Hippo pathway (e.g., with verteporfin) significantly reduces fibrosis‐related genes such as COL1A1 and α‐SMA, highlighting a potential therapeutic strategy.

In DD, SFRP4 promotes persistent inflammation and fibrosis through NF‐κB and STAT3 signaling. Mechanistically, it interacts with β‐TrCP and IκBα, facilitates IκBα degradation, activating NF‐κB in palmar fascia fibroblasts [250]. Silencing SFRP4 via siRNA reduces fibroblast proliferation, migration, and myofibroblast activation, while nanoparticle‐delivered SFRP4 siRNA suppresses fibrosis in a DD‐graft mouse model [251], suggesting a promising therapeutic target.

Unlike SFRP2 and SFRP4, SFRP1 appears protective against fibrosis. It is downregulated in keloid fibroblasts, likely due to decreased histone acetylation [233] and increased DNA methylation [244]. Overexpression of SFRP1 in keloid fibroblasts reduces β‐catenin and α‐SMA levels [244], suggesting an inhibitory effect on Wnt/β‐catenin signaling and scar formation.

In oral wound healing, a process known for rapid repair with minimal scarring [286, 287], SFRP1 and SFRP5 are highly expressed. However, in oral submucous fibrosis, their levels decline due to promoter hypermethylation, leading to excessive Wnt/β‐catenin signaling and fibrosis progression [252, 253].

### 5.2 SFRPs in Cardiovascular Fibrosis

Unlike skin fibrosis, SFRPs in the cardiovascular system exhibit more distinct regulatory patterns. SFRP1, SFRP3, and SFRP5 exert protective effects against cardiovascular fibrosis by mitigating inflammation and reducing collagen deposition following myocardial injury. In contrast, SFRP2 and SFRP4 exhibit dual roles, acting as both profibrotic and antifibrotic regulators depending on the context.

SFRP1 protects against cardiac fibrosis primarily by inhibiting the Wnt/β‐catenin pathway. It reduces TGF‐β1‐induced cardiac fibroblast proliferation, collagen synthesis, and myofibroblast activation while promoting apoptosis [254]. In aged mice with acute myocardial ischemia, SFRP1 overexpression suppressed myocardial fibrosis, reduced cardiomyocyte apoptosis, and improved cardiac function. These effects were confirmed in SFRP1 knockout mice and patients with dilated cardiomyopathy and ischemic dilated cardiomyopathy [254, 257, 288].

Like SFRP1, SFRP3 exerts cardioprotective effects by reducing fibrosis in ischemia/reperfusion injury and slowing heart failure progression [289]. Its overexpression in cardiomyocytes alleviates Ang II‐induced hypertrophy, fibrosis, oxidative stress, and apoptosis [260]. Conversely, SFRP3 downregulation following myocardial infarction (MI) promotes cardiac fibrosis by inducing EndMT through FOXM1‐mediated activation of Wnt/β‐catenin signaling [265].

SFRP5 downregulation, mediated by the increased level of miR‐125b, contributes to CF activation, proliferation, migration, and increased expression of α‐SMA and collagen I/III in patients with acute MI [255, 256]. In contrast, AAV‐mediated SFRP5 overexpression significantly improves left ventricular function and reduces hypertrophy by suppressing cardiac fibrosis, partially via AMPK pathway activation in the MI mouse model [262]. Similarly, in type 2 diabetes mellitus models of MI, SFRP5 treatment mitigates fibrosis and improves cardiac function by inhibiting the Wnt5a/JNK pathway [264].

SFRP2 exhibits potent antifibrotic effects in therapeutic settings. In a rat model of MI, both endogenous SFRP2 at high concentration and injected recombinant SFRP2 reduce MI‐induced fibrosis, prevent anterior wall thinning [229]. Moreover, SFRP2 enhances angiogenesis via endoplasmic reticulum stress activation, particularly through transcription factor 6 (ATF6), and promotes vascular growth via CTGF signaling [230]. In addition, it enhances mesenchymal stem cells (MSCs) engraftment, serving as a key paracrine factor in Akt‐modified MSCs by promoting hypoxic cardiomyocyte survival and facilitating myocardial repair [258, 259, 263].

However, SFRP2 can also contribute to fibrosis by modulating ECM dynamics. In CFs, it regulates collagen formation and ECM degradation by altering BMP1 activity in a concentration‐dependent manner [229, 259, 266]. Furthermore, SFRP2 has been shown to activate Wnt/β‐catenin pathway to promote CFs proliferation via increased anaerobic glycolysis, marked by elevated levels of tissue nonspecific alkaline phosphatase (TNAP) and glyceraldehyde‐3‐phosphate dehydrogenase (GAPDH) activity [266]. These findings highlight its dual role in cardiac remodeling, where its effects vary depending on the microenvironment and disease stage.

The role of SFRP4 in cardiovascular fibrosis remains controversial. Zeng et al. reported that SFRP4 knockdown activated the PI3K/AKT pathway, thereby reducing cardiomyocyte apoptosis, decreasing infarct size, and improving cardiac function following myocardial ischemia/reperfusion injury [267]. In contrast, Matsushima et al. found that intramyocardial delivery of recombinant SFRP4 protein conferred dose‐dependent cardioprotection, enhancing postinjury cardiac function and suggesting an antifibrotic potential in cardiac ischemia [261].

### 5.3 SFRPs in Pulmonary Fibrosis

Moving beyond cardiovascular functions, SFRPs reveal a distinct regulatory landscape within pulmonary fibrosis. SFRP1 plays complex and context‐dependent roles in lung injury repair and fibrosis. Several studies have reported that SFRP1 expression is significantly downregulated in pulmonary fibroblasts, in response to TGF‐β1, EGF, and FGF2. This downregulation promotes fibroblast invasion by increasing RhoA activity [268]. In pulmonary fibrosis, hypermethylation‐induced silencing of SFRP1 correlates with increased disease severity, while treatment with a DNA methyltransferase inhibitor 5‐aza‐2′‐deoxycytidine (5‐aza) restores their expression and improves lung function [237], suggesting a protective role for SFRP1. However, Lories et al. found that while SFRP1 reduced TGF‐β1‐induced collagen upregulation in vitro, its absence did not significantly affect fibrotic responses in vivo, suggesting possible functional redundancy [290]. These discrepancies may stem from differences in fibrosis assessment and warrant further investigation.

Adding to the complexity, extracellular vesicles (EVs) from bronchoalveolar lavage fluid (BALF) of mouse models have implicated SFRP1 in exacerbating fibrosis. EVs from BAL have been implicated in pulmonary fibrosis, with SFRP1‐enriched EVs exacerbating disease progression. In murine and human lung fibrosis, fibroblasts, which are the key effector cells driving ECM deposition, are also the predominant source of EVs. These vesicles carry profibrotic mediators such as Wnt5a that further enhance fibroblast activation. Due to their distinct cargo and biomarker potential, BALF‐EVs represent promising tools for diagnosis, prognosis, and therapeutic targeting in pulmonary fibrosis [291]. Mice receiving EVs from SFRP1‐deficient fibroblasts exhibited significantly less lung collagen accumulation than those receiving EVs from wild‐type fibroblasts [270]. This apparent contradiction with SFRP1's previously suggested antifibrotic role underscores the need for a more comprehensive analysis of its function in pulmonary fibrosis. Beyond its role in fibroblasts regulation, SFRP1 also maintains the undifferentiated state of bronchoalveolar stem cells and small airway epithelial cells during lung injury [269], supporting tissue repair and regeneration.

SFRP2 and SFRP4 are also implicated in pulmonary fibrosis, playing distinct roles at different stages of the disease. Single‐cell RNA sequencing revealed that SFRP2 is induced by proinflammatory cytokines (e.g., IL‐1, TNF) and contributes to early‐stage inflammation by promoting immune cell recruitment. In contrast, SFRP4 is upregulated by interferons and functions in later‐stage fibrosis, facilitating tissue repair. Spatial analysis of IPF lung tissues further supports this distinction, showing that SFRP2^+^ fibroblasts localize at the edges of fibrotic lesions, while SFRP4^+^ fibroblasts are more abundant within the lesions, particularly in interstitial areas [292]. These findings underscore the spatial and temporal dynamics of SFRP2 and SFRP4 in lung inflammation and fibrosis.

### 5.4 SFRPs in Liver Fibrosis

Levels of SFRPs in fibrotic livers are elevated compared with normal ones; however, this does not necessarily indicate a profibrotic role. Instead, it may reflect a compensatory response to counteract excessive Wnt signaling. Wang et al. demonstrated that inhibiting SFRP1 with Sja‐miR‐1, a microRNA derived from *Schistosoma japonicum*, activated Wnt/β‐catenin signaling in HSCs, the liver's fibroblasts. This activation upregulated fibrosis‐associated genes, including α‐SMA, Col1α1, and Col3α1, thereby promoting liver fibrosis [271]. Similarly, Myung et al. found that Wnt3a increased the expression of fibrotic markers (Col1α1 and α‐SMA) and suppressed TRAIL‐mediated apoptosis in human HSCs, while SFRP1 overexpression mitigated these effects, reducing fibrosis marker expression and enhancing apoptosis [272].

SFRP5 appears to function similarly to SFRP1 in liver fibrosis. Recombinant SFRP5 at concentration of 2 µg/mL inhibited murine HSC proliferation and migration by blocking Wnt5a/Fz2 signaling and reducing JNK phosphorylation [273]. In contrast, SFRP5 deficiency exacerbated CCl_4_‐induced liver fibrosis by enhancing Wnt5a–JNK signaling. These findings suggest that SFRPs, particularly SFRP1 and SFRP5, may serve as protective regulators in liver fibrosis by modulating Wnt signaling and HSC activity.

### 5.5 SFRPs in Renal Fibrosis

In renal fibrosis, SFRPs drive fibrogenesis through mechanisms distinct from those in liver fibrosis. SFRP1 and SFRP5 play pivotal roles in renal fibrosis, though their mechanisms remain complex.

Noncoding RNAs, including lncRNAs and miRNAs, have also emerged as key regulators and potential therapeutic targets in renal pathophysiology, influencing conditions such as acute kidney injury, chronic kidney disease, diabetic nephropathy, glomerular diseases, and renal cancers, while serving as prognostic biomarkers [293]. For example, in a rat model of diabetic nephropathy, elevated glucose levels induced miRNA‐27a expression, suppressing SFRP1, activated the Wnt/β‐catenin pathway, and promoted fibrosis [274]. In contrast, studies in SFRP1 knockout mice revealed increased phosphorylation of c‐Jun and JNK in obstructed kidneys, suggesting that SFRP1 may contribute to fibrosis via noncanonical Wnt/PCP signaling [275]. These seemingly contradictory findings may arise from differences in experimental models, underscoring the need for further investigation into SFRP1's role in renal fibrosis.

SFRP5, however, consistently exerts protective effects. Its overexpression inhibited Wnt/β‐catenin signaling and suppressed epithelial‐to‐mesenchymal transition (EMT) in both a rat model of unilateral ureteral obstruction‐induced renal fibrosis and human proximal tubular epithelial HK‐2 cells [276]. Similarly, hypermethylation of the SFRP5 gene was observed in renal tubular cells and a mouse model of indoxyl sulfate‐driven chronic kidney disease, leading to reduced SFRP5 expression, Wnt/β‐catenin activation, and fibrosis progression [238].

Overall, SFRPs exhibit diverse and context‐dependent roles in fibrosis across the lung, liver, and kidney, influencing both inflammatory responses and tissue repair. Further research is essential to clarify their therapeutic potential. An overview is provided in Figure 6.

### 5.6 SFRPs in Immune‐Related Fibrotic Disorders

Fibrosis is a major complication in immune‐related diseases such as SSc and RA. Emerging evidence highlights SFRPs as key modulators of fibrotic processes in these conditions.

In SSc, hypermethylation of the SFRP1 promoter leads to reduced expression in fibroblasts and peripheral blood mononuclear cells, resulting in overactivated Wnt signaling and exacerbating fibrosis. Treatment with the methyltransferase inhibitor 5‐aza attenuates canonical Wnt signaling and alleviates fibrosis in both SSc fibroblasts and a bleomycin‐induced mouse model, underscoring SFRP1's protective role [239].

scRNA‐seq analysis identified a fibroblasts subpopulation coexpressing SFRP2 and SFRP4, which correlated with increased skin fibrosis severity in SSc patients. These fibroblasts were linked to tissue remodeling and collagen fiber organization, suggesting a profibrotic role [277]. Further studies revealed that SSc dermal myofibroblasts arise from an SFRP2^hi^CD26^+^ progenitor population through a two‐step differentiation process. Only a subset of these SFRP2^hi^ fibroblasts transition into myofibroblasts, marked by SFRP4 and FNDC1 expression [278]. This transition appears critical in SSc progression, though whether SFRP4 and SFRP2 drive or result from fibrosis remains unclear.

In RA, all SFRPs are markedly downregulated in synovial fibroblasts, suggesting a protective role [279, 282, 284]. SFRPs suppress fibrotic genes like fibronectin and LRP5 by inhibiting Wnt/β‐catenin signaling while also reducing proinflammatory mediators (IL‐1β, IL‐6, and MMP9) via JNK inhibition [284]. This dual action mitigates fibroblasts adhesion, migration, and inflammation [282]. In a rat model of collagen‐induced arthritis, promoter hypermethylation led to decreased SFRP2 and SFRP4 expression, while treatment with 5‐aza restored their levels, curbing synovial fibroblasts proliferation and inflammation [280, 283]. Additionally, TNFα‐driven activation of enhancer of zeste homolog 2 (EZH2) via the NF‐κB/JNK pathway resulted in SFRP1 silencing, sustaining chronic activation of synovial fibroblasts and disease progression [279]. Restoring SFRP1 (via NSUN2 silencing) or SFRP2 (using the hesperidin derivative HDND‐11) effectively suppressed Wnt/β‐catenin signaling and halted RA progression [281, 294].

Collectively, these findings highlight SFRPs as pivotal regulators in immune‐related fibrosis, with potential therapeutic implications for both SSc and RA.

## 6 Therapeutic Targeting Of Wnt Signaling Pathway In Tissue Fibrosis

Aberrant Wnt signaling is a key driver of fibrotic diseases across multiple organs, making it an attractive therapeutic target. Dysregulation of core Wnt components and their crosstalk with other pathways not only promote fibrosis but are also implicated in cancer, where most Wnt‐targeted therapies have been developed. Notably, several first‐line cancer treatments also exert antifibrotic effects. Growing numbers of preclinical studies are now exploring Wnt inhibition in fibrosis, and clinical trials are beginning to test the safety and efficacy of these approaches. Table 2 summarizes preclinical Wnt‐targeted antifibrotic studies across organ systems, while Table 3 outlines ongoing clinical trials of Wnt pathway modulation. Together, these data indicate that Wnt inhibition shows robust antifibrotic efficacy in preclinical models, largely by suppressing β‐catenin activity, myofibroblast activation, and ECM deposition. However, most strategies remain confined to animal studies, reflecting challenges related to pathway pleiotropy, dosing, and off‐target effects. Clinically, Wnt‐targeted therapies have largely transitioned from oncology into early‐phase trials, with PRI‐724 among the few agents directly evaluated in fibrotic diseases, highlighting both the translational promise of Wnt modulation and the need for more selective, context‐adapted approaches to ensure safety. The details are discussed in the following subsections.

**TABLE 2:** Preclinical Wnt‐targeted antifibrotic studies.

Disease	Therapy	Species	References
Pulmonary fibrosis	XAV939	R	[295]
	Calcaratarin D	M	[296]
	Physalis Calyx seu Fructus	M	[297]
Muscle fibrosis	Ginsenoside Rb1	R	[298]
Hepatic fibrosis	Taraxasterol	M	[299]
	Cordycepin	M	[300]
	Doublecortin domain containing 2	M	[301]
	Gandouling	M	[302]
Renal fibrosis	ADSC‐EV	M	[303]
	FGF21	M	[304]
	Oridonin	R	[305]
Gastric fibrosis	Genistein	R	[306]

Abbreviations: ADSC‐EV, adipose mesenchymal stem cells derived extracellular vesicles; FGF21, fibroblast growth factor 21; M, mice; R, rat.

**TABLE 3:** Clinical trials of Wnt pathway modulation.

Disease	Main drug	Target	Phase	NCT number
Metastatic colorectal cancer	Genistein	Wnt/β‐catenin	I/II	NCT01985763
Advanced solid tumors	RXC004	PORCN	I	NCT03447470
Recurrent epithelial endometrial cancer, epithelial ovarian cancer, or carcinosarcoma	DKN‐01	DKK1	II	NCT03395080
Moderately to severely symptomatic knee osteoarthritis	SM04690	CLK2/DYRK1A	II	NCT03122860
Pediatric relapsed/refractory acute leukemia	Daunorubicin	β‐Catenin/Akt	II	NCT04562792
Liver fibrosis	PRI‐724	CBP/β‐catenin	I/II	NCT03620474

*Data source*: International Clinical Trials Registry Platform (https://trialsearch.who.int/), accessed on September 22, 2025.

### 6.1 Small Molecules and Biologic Inhibitors of Wnt Signaling

Pharmacologic disruption of Wnt signaling has yielded several classes of inhibitors that are currently under preclinical and clinical development. Among small‐molecule inhibitors, PORCN inhibitors block the membrane‐bound acyltransferase PORCN, thereby preventing secretion of all Wnt ligands. The tankyrase inhibitors, act by stabilizing AXIN, which enhances β‐catenin degradation. XAV‐939, a representative compound, restores β‐catenin turnover and reduces ECM accumulation in pulmonary fibrosis [295].

Another strategy involves disrupting β‐catenin's transcriptional activity. The β‐catenin/CBP inhibitor PRI‐724 prevents the interaction between β‐catenin and the coactivator CBP, thereby blocking fibrogenic transcriptional programs. PRI‐724 reduces collagen deposition in IPF and liver fibrosis models and is currently in Phase II trials for myelofibrosis and IPF [307]. Natural compounds have also demonstrated antifibrotic potential through Wnt modulation. For instance, ginsenoside Rb1 downregulates Wnt3/β‐catenin signaling to inhibit EMT in lung fibroblasts [298], while taraxasterol attenuates CCl_4_‐induced liver fibrosis through coordinated regulation of the Wnt, Hippo, HIF‐1α, and TGF‐β/Smad pathways [299].

In parallel, biologic inhibitors have been developed to more selectively target Wnt ligands or receptors. Monoclonal antibodies against Wnt3a block canonical Wnt activity and can redirect signaling toward noncanonical pathways, thereby reducing myofibroblast differentiation [308, 309]. Similarly, functional knockdown of Fz7 suppresses TGF‐β1‐induced expression of α‐SMA, collagen I, fibronectin, and CTGF, ultimately attenuating pulmonary fibrosis in vivo [310]. Additional biologic strategies include the use of soluble decoy receptors or engineered ligands that sequester Wnt ligands. Some of these approaches function as “paradoxical agonists”, shifting signaling from canonical to noncanonical branches in order to blunt profibrotic outcomes. Collectively, these small‐molecule and biologic inhibitors illustrate the versatility of targeting Wnt signaling, ranging from broad suppression of ligand secretion to precision modulation of ligand–receptor interactions.

### 6.2 SFRP‐Based Modulation of Wnt Activity

As endogenous antagonists of Wnt signaling, SFRPs modulate pathway activity by sequestering Wnt ligands or competing with Fz receptors, thereby restraining β‐catenin‐dependent transcription. Across organ systems, restoring SFRP function via recombinant proteins, mimetic peptides, or epigenetic reactivation has shown antifibrotic efficacy. These strategies consistently reduce hallmark fibrotic markers (α‐SMA, collagens I/III), limit fibroblast activation and proliferation, and improve tissue architecture and function [28].

Proof‐of‐concept studies illustrate multiple translational routes. Recombinant protein therapy with SFRP4 ameliorated renal fibrosis in unilateral ureteral obstruction models by dampening tubular epithelial β‐catenin signaling [311, 312]. Epigenetically, promoter hypermethylation contributes to SFRP silencing in fibrotic disease. Treatment with demethylating agents such as 5‐azacytidine restored SFRP1 expression and exerted antifibrotic effects in pulmonary fibrosis and SSc models [237, 239]. Similarly, in post‐MI fibrosis, angiotensin receptor–neprilysin inhibitor therapy improved cardiac function in part by increasing SFRP1 expression [313].

### 6.3 Limitations and Challenges in Translating SFRP‐Based Therapies

Despite their promising antifibrotic potential, therapeutic strategies targeting SFRPs remain underexplored and face significant translational barriers. As secreted glycoproteins, SFRPs primarily inhibit Wnt ligand–Fz receptor interactions, a process that is structurally and mechanistically difficult to modulate with small molecules. Consequently, most current approaches rely on larger biologics such as monoclonal antibodies and recombinant proteins. While these agents show efficacy in preclinical settings, they are hindered by inefficient delivery into fibrotic tissues, high production costs, and complex manufacturing processes. Tools such as antibodies and siRNAs have so far remained largely restricted to basic research, and their clinical application will require improved tissue penetration strategies as well as rigorous safety validation. Innovative screening platforms, including organs‐on‐chips [314] and organoid [315] systems that more closely replicate in vivo environments, may accelerate optimization and translational readiness.

Another critical challenge is safety and specificity. Systemic administration of anti‐SFRP antibodies or recombinant proteins carries the risk of off‐target immune responses and unintended disruption of homeostatic Wnt activity in nonfibrotic tissues. Achieving organ‐ and context‐specific modulation of SFRP activity will therefore be essential for clinical viability.

Despite substantial progress, key mechanistic gaps remain. The biphasic regulation of SFRPs, their context‐dependent interactions with Wnt ligands, and the distinct contributions of individual family members across tissues and pathological states are still incompletely understood. For instance, recent studies highlight the skin fascia as a critical contributor of wound repair and scar formation [316]. Fascia fibroblasts undergo collective migration while transitioning through distinct cellular states [286], from CD201^+^ progenitors to PDPN^+^ inflammatory fibroblasts, pSTAT3^+^ proto‐myofibroblasts, and eventually RUNX2^+^ myofibroblasts [317]. Within this trajectory, single‐cell RNA sequencing has revealed an SFRP2‐expressing fibroblast subset, yet its precise role in fascia mobilization, ECM remodeling, and wound healing remains unexplored. Elucidating how SFRP2 shapes fibroblast transitions could unlock new therapeutic strategies for both wound repair and scar management.

## 7 Conclusions And Future Perspectives

In summary, this review highlights the central role of Wnt signaling across diverse physiological and pathological processes, with particular focus on its involvement in tissue fibrosis, and special emphasis on the critical functions of SFRPs. Far from being simple antagonists, SFRPs act as versatile modulators whose effects are determined by cellular context, fibroblast state, and organ environment. While most family members restrain canonical Wnt activity and thereby exert antifibrotic functions, accumulating evidence shows that they can also act as agonists or facilitators of fibrosis under specific conditions. This duality underscores both the complexity of SFRP biology and its therapeutic potential.

Emerging single‐cell RNA sequencing and spatial transcriptomics have unveiled fibroblast heterogeneity in fibrotic tissues, identifying distinct SFRP‐expressing subsets that contribute to disease progression or resolution. Furthermore, the biphasic effects of SFRPs, such as SFRP1's antifibrotic role in cardiac and renal fibrosis versus its profibrotic action in pulmonary EV‐mediated signaling, highlight the need for precision therapeutics tailored to organ‐specific contexts [254, 270, 275]. Recent preclinical breakthroughs, including the use of epigenetic modulators and biologics, demonstrate translational potential.

From a translational perspective, advancing SFRP‐based therapies toward clinical application will require several concrete and coordinated steps. Current therapeutic strategies largely rely on recombinant SFRP proteins or monoclonal antibodies that modulate Wnt ligand–receptor interactions; however, their clinical efficacy is constrained by limited in vivo stability, short half‐life, and challenges in tissue‐specific delivery. Future efforts should therefore prioritize the development of optimized biologics, such as engineered SFRP variants with enhanced stability, long‐acting formulations, or targeted delivery systems (e.g., nanoparticle‐ or EV‐based carriers) to improve bioavailability and organ specificity. In parallel, epigenetic modulators that restore endogenous SFRP expression, for example, through DNA methylation or chromatin remodeling pathways, represent an attractive complementary strategy. Successful translation will also depend on the identification and validation of robust biomarkers, such as SFRP expression signatures, Wnt activity states, or fibroblast subtype markers, to enable patient stratification and therapeutic monitoring. Given the context‐dependent and dose‐sensitive nature of SFRP function, rational combination therapies that fine‐tune rather than globally suppress Wnt signaling may be essential to preserve tissue homeostasis while achieving antifibrotic efficacy.

Consequently, future directions should focus on these key directions: elucidating underlying mechanisms, exploring crossorgan insights, and advancing preclinical/clinical translation. More precisely, efforts could be directed toward further elucidating the molecular determinants of SFRP duality, including structural interactions, posttranslational modifications, and crosstalk with other pathways (e.g., TGF‐β, Hippo) in tissue fibrosis. Given the conserved nature of certain fibrotic mechanisms such as fascia fibroblast mobilization alongside the context‐dependent effects of SFRPs, it is promising to pursue pan‐fibrotic targets while addressing organ‐specific subtle distinctions. Translational efforts should emphasize the development of SFRP‐based biomarkers for patient stratification, alongside preclinical testing of biologics and epigenetic modulators targeting Wnt‐SFRP signaling.

Altogether, a deeper understanding of Wnt regulation through SFRPs offers not only conceptual insights into fibroblast heterogeneity but also a promising path toward precision therapies for fibrotic diseases.

## Author Contributions

Writing – original draft preparation, X.J., J.W., R.C., and D.J. Writing – review and editing: X.J., J.W., R.C., and D.J. Funding acquisition: D.J. All authors have read and approved the final manuscript.

## Ethics Statement

The authors have nothing to report.

## Conflicts of Interest

The authors declare no conflicts of interest.

## Data Availability

The authors have nothing to report.
